# VisualRNet: Lightweight Camera Rotation Estimation from Low-Resolution Optical Flow via Cross-Modal Supervision

**DOI:** 10.3390/s26092655

**Published:** 2026-04-24

**Authors:** Xiong Yang, Hao Wang, Jiong Ni

**Affiliations:** 1 School of Cyberspace Security, Changzhou College of Information Technology, Changzhou 213164, China; 2 School of Computer Science, Xi’an Jiaotong University, Xi’an 710049, China

**Keywords:** camera rotation estimation, optical flow, lightweight neural network, cross-modal supervision, few-shot adaptation

## Abstract

Camera rotation estimation is a key component in video stabilization and motion analysis. In many practical scenarios, inertial measurements are unavailable or temporally unreliable, while classical geometric pipelines degrade under blur, low texture, and low illumination. This paper investigates whether substantially downsampled optical flow can retain sufficient structure for accurate frame-to-frame rotation regression. We present VisualRNet, a lightweight rotation-specific visual regression framework trained with cross-modal IMU supervision. Our design uses coordinate-aware feature encoding, depthwise separable convolutions, lightweight attention, and a compact 6D rotation head to model the spatial structure of rotational flow fields. On Deep-FVS, VisualRNet achieves a mean rotation error of 0.3151° on the test set. The VisualRNet regression head contains 7.7 K parameters, 0.002 GFLOPs, and runs at 729 FPS, while the full pipeline with the FastFlowNetv2 frontend contains 1.374 M parameters, 7.194 GFLOPs, and runs at approximately 113 FPS. A cross-camera adaptation experiment on TUM VI further indicates that the learned motion representation can be aligned to a new camera system with limited calibration data. These results support low-resolution optical flow as a practical input for visual rotation estimation and suggest particular value in stabilization-oriented and cost-sensitive applications where approximate rotational trend matters more than full scene geometry.

## 1. Introduction

Camera rotation estimation is a core task in ego-motion perception and a practical sensing component for electronic image stabilization (EIS), visual odometry (VO), augmented reality (AR), and robotic navigation. In ideal hardware settings, rotational states are measured by micro-electromechanical system (MEMS) inertial measurement units (IMUs). In practice, however, inertial data may be absent from legacy video, omitted in low-cost devices, or affected by synchronization offsets and temporal drift relative to the image stream. These limitations motivate purely visual approaches that can infer rotational states directly from image motion.

Classical visual solutions typically rely on local feature detection, descriptor matching, and robust geometric fitting, for example through ORB and RANSAC [1,2]. Although mathematically well grounded, such pipelines are fragile in low-texture scenes and degraded imaging conditions, where feature extraction and matching often fail. Learning-based visual odometry has shown that camera motion can also be regressed directly from video [3,4,5]. However, many deep RGB-based systems are designed for broader 6 DoF ego-motion estimation and therefore carry substantially higher appearance-processing cost than is necessary for stabilization-oriented rotational sensing.

This paper focuses on a narrower question: can frame-to-frame camera rotation be estimated accurately from low-resolution optical flow? Our motivation is that rotational cues are often reflected in the global spatial structure of optical flow, whereas many appearance details in RGB images are irrelevant to this sub-task. We therefore reformulate the problem as a motion-centric regression task. Instead of treating the network as a generic image recognizer, we inject normalized spatial coordinates through CoordConv so that the model can interpret the flow field relative to the image center and better exploit the spatial organization induced by camera rotation.

Based on this formulation, we design **VisualRNet**, a compact regression network that operates on 80×45 optical flow and predicts frame-to-frame camera rotation in a continuous 6D representation. During training, synchronized IMU measurements provide cross-modal supervision; during inference, the model serves as a purely visual rotation estimator. Our experiments show that this compact representation is effective on Deep-FVS, and that the learned motion features can be adapted to a new camera system with limited calibration data on TUM VI. Importantly, the lightweight statistics emphasized in this paper refer to the rotation regression head itself; the end-to-end system additionally includes the optical-flow frontend.

The main contributions of this paper are summarized as follows:

- We present VisualRNet, a rotation-specific visual regression framework that estimates frame-to-frame camera rotation directly from low-resolution optical flow under cross-modal IMU supervision.
- We design a compact coordinate-aware architecture that combines low-resolution motion input, CoordConv, lightweight convolutional blocks, and a continuous 6D rotation head, and we validate the role of these components through ablation studies.
- We show on Deep-FVS that the proposed method provides accurate and efficient rotation estimation, and we further demonstrate its cross-camera adaptability through lightweight few-shot calibration on TUM VI.

## 2. Related Work

### 2.1. Classical Geometric Rotation Estimation

Relative camera motion has long been studied through epipolar geometry, feature matching, and robust model fitting [6,7,8,9]. Such methods remain important baselines, but their stability depends on the availability of repeatable correspondences. In difficult conditions, including motion blur, low illumination, or weak texture, these assumptions are frequently violated. Our work is motivated by these practical failure modes and targets a lightweight visual alternative for rotation sensing rather than a replacement for full geometric VO pipelines.

### 2.2. Learning-Based Visual Motion Estimation

Deep learning has enabled the direct regression of camera motion from image sequences [3,4,5,10]. These methods typically address broader pose-estimation settings and often process high-resolution RGB appearance, which increases model complexity and can entangle motion estimation with scene appearance. In contrast, VisualRNet deliberately focuses on the rotation sub-problem and uses optical flow as the sole input, allowing the network to operate on a much more compact motion representation.

### 2.3. Broader Feed-Forward Visual Geometry Models

Recent visual geometry systems such as DUSt3R, MASt3R, VGGT, and Align3R aim to infer richer scene-level quantities, including camera parameters, point maps, depth maps, feature tracks, or temporally aligned video geometry, often from uncalibrated or weakly calibrated imagery [11,12,13,14]. These capabilities are important when the downstream goal is scene reconstruction or general 3D reasoning. Our objective is deliberately narrower: we do not attempt to recover full scene geometry, absolute depth, or multi-view structure, but instead target a lightweight motion-sensing module for frame-to-frame rotation estimation when approximate rotational trend and magnitude are sufficient for downstream processing.

### 2.4. Optical Flow as a Motion-Centric Representation

Modern optical-flow networks such as FlowNet, PWC-Net, RAFT, and FastFlowNet have made dense motion estimation practical in real time [15,16,17,18]. Most prior work uses flow as an intermediate cue for downstream tasks. We instead investigate whether low-resolution flow alone is sufficient for frame-to-frame rotation regression. This perspective is also related to direct motion-parameter reconstruction lines that infer global motion descriptors from image dynamics without relying on sparse feature correspondences [19,20,21]. These works show that motion fields can carry explicit motion parameters for tracking and stabilization. Our method differs in that it learns a compact rotation regressor from dense flow produced by a modern optical-flow frontend and is trained under cross-modal supervision for frame-to-frame camera rotation estimation.

### 2.5. Rotation Sensing for Video Stabilization

Video stabilization relies heavily on reliable estimates of camera motion [22,23,24,25]. In this context, frame-to-frame rotation is especially important because rotational jitter is a dominant source of perceived shakiness. VisualRNet is positioned as a lightweight visual sensing module for such stabilization-oriented settings: it does not aim to solve full 6 DoF ego-motion, but rather to provide accurate rotational estimates from motion cues when inertial data are unavailable or unreliable.

## 3. Materials and Methods

### 3.1. Problem Formulation and Coordinate Systems

In this study, we estimate the relative rotation R∈SO(3) between two consecutive video frames $I_{t}$ and $I_{t+1}$. We deliberately focus on frame-to-frame camera rotation rather than full 6 DoF motion, because rotational cues are particularly important for stabilization-oriented applications and can be more directly inferred from the global structure of optical flow. Dense optical flow is first computed between the two frames and then downsampled to a low spatial resolution to reduce computational cost and suppress high-frequency noise.

Let $F\in R^{2\times H^{\prime }\times W^{\prime }}$ denote the optical-flow input, where $H^{\prime }=45$ and $W^{\prime }=80$. The two channels correspond to the horizontal and vertical flow components. Here, we use the term “optical-flow tensor” to refer to the array representation fed into the network, while the underlying physical quantity remains a 2D vector field defined on the image plane. Our objective is to learn a mapping Φ:F→R that regresses the 3 DoF rotational state directly from this compact motion representation.

Although strong downsampling discards fine semantic detail, we hypothesize that the global spatial organization induced by camera rotation is largely preserved. VisualRNet is therefore designed to exploit this low-resolution motion structure rather than appearance content, with the image-center coordinates injected explicitly so that the network can interpret the flow field relative to the camera geometry.

### 3.2. Coordinate-Aware Geometric Representation

The image motion induced by camera rotation is spatially structured: the direction and magnitude of flow vary systematically with pixel position relative to the image center. In this paper, the term “coordinate-aware” is used in an engineering sense to describe a coordinate-aware inductive bias rather than a formally group-equivariant network design. By injecting normalized (x,y) coordinates through CoordConv, we allow the model to interpret identical flow values differently depending on where they occur in the image. This makes it easier for the network to exploit the center-referenced spatial organization of rotational flow fields.

### 3.3. Cross-Modal Supervision via Sensor Fusion

**Figure 1:**
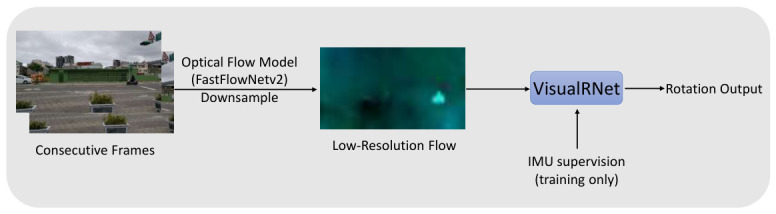
Overall pipeline of the proposed framework. Two consecutive RGB frames are first processed by an efficient optical-flow frontend based on FastFlowNetv2 and then downsampled to a low spatial resolution. The resulting compact, low-resolution optical flow field serves as the sole input to VisualRNet for frame-to-frame camera rotation estimation. During training, synchronized IMU measurements provide cross-modal supervision; during inference, the IMU is not used.

To overcome the lack of directly annotated visual rotation labels in real-world videos, our framework adopts a cross-modal supervision paradigm, as illustrated in the overall pipeline in Figure 1. During training, we leverage synchronized inertial measurements from a Micro-Electro-Mechanical System (MEMS) IMU. These signals provide physically grounded supervisory information that is independent of visual degradations such as motion blur, illumination changes, or lens flare.

More specifically, the network is trained against an IMU-derived reference rotation constructed from the gyroscope signal over the corresponding frame interval. This can be interpreted as cross-modal knowledge transfer: during training, inertial measurements supervise the visual regressor; during inference, the IMU is not used, and VisualRNet functions as a compact visual rotation-sensing module for settings in which inertial data are unavailable, drift-prone, or temporally unsynchronized.

### 3.4. Dataset Characteristics and Preprocessing

We conduct experiments on the Deep-FVS dataset [26], which contains diverse hand-held camera trajectories in indoor and outdoor scenes. As the optical-flow frontend, we use the FastFlowNetv2 architecture [18] and implement it through a v2-compatible code path in our current environment. This preserves the original FastFlowNet design while ensuring compatibility with our PyTorch 2.6.0 and CUDA 12.4 setup. The extracted flow fields are normalized before being fed into the rotation regressor.

Standard spatial augmentations such as random cropping, flipping, or arbitrary image rotation are avoided. Since the target labels describe camera rotation relative to the original image coordinates, such transformations would alter the spatial correspondence between the flow field and the synchronized inertial supervision. We follow the official data split and report results on 19,363 test samples.

### 3.5. Continuous 6D Rotation Representation

The mathematical representation of rotation is critical for stable learning. Euler angles suffer from singularities, while quaternions exhibit antipodal ambiguity. Following Zhou et al. [27], we therefore adopt a continuous 6D representation of SO(3).

The regression head outputs a 6D vector $\hat{r}=\left[r_{1},\ldots ,r_{6}\right]\in R^{6}$, which is split into two unconstrained 3D vectors,

$$a_{1}={\left[r_{1},r_{2},r_{3}\right]}^{⊤},\;a_{2}={\left[r_{4},r_{5},r_{6}\right]}^{⊤}.$$

These vectors are converted into an orthonormal basis through a differentiable Gram–Schmidt process:

(1) $$b_{1}=\frac{a_{1}}{∥a_{1}{∥}_{2}},\;b_{2}=\frac{a_{2}-\left(b_{1}\cdot a_{2}\right)b_{1}}{∥a_{2}-\left(b_{1}\cdot a_{2}\right)b_{1}{∥}_{2}},\;b_{3}=b_{1}\times b_{2}$$

The final rotation matrix is then formed as $R=\left[b_{1},b_{2},b_{3}\right]\in SO\left(3\right)$. This continuous parameterization improves optimization stability and avoids discontinuities in the target space.

### 3.6. Geodesic Loss on the SO(3) Manifold

To provide a mathematically rigorous supervision signal, we move beyond the standard Mean Squared Error (MSE), which merely treats the matrix as an independent 9-element array. Instead, we employ the geodesic distance on the SO(3) manifold as our primary optimization objective [6]. This loss directly measures the minimum physical angle required to rotate the predicted camera coordinate frame into alignment with the reference orientation.

Given the predicted rotation $R_{\mathrm{pred}}$ and the reference rotation $R_{\mathrm{ref}}$, we first compute the relative error matrix $R_{\mathrm{rel}}=R_{\mathrm{pred}}^{⊤}R_{\mathrm{ref}}$. According to the angle-axis representation, the trace of a rotation matrix is directly related to its rotation angle θ via the identity trace(R)=1+2cos(θ). Thus, the geodesic loss is defined as in Equation (2):

(2) $$L_{geo}=\arccos\left(\frac{\mathrm{trace}(R_{\mathrm{rel}})-1}{2}\right)$$

By directly optimizing for this geodesic distance, the network is directly incentivized to minimize the absolute angular error in degrees, offering a more robust and physically meaningful gradient than element-wise matrix regression.

### 3.7. VisualRNet: A Principled Architectural Path

**Figure 2:**
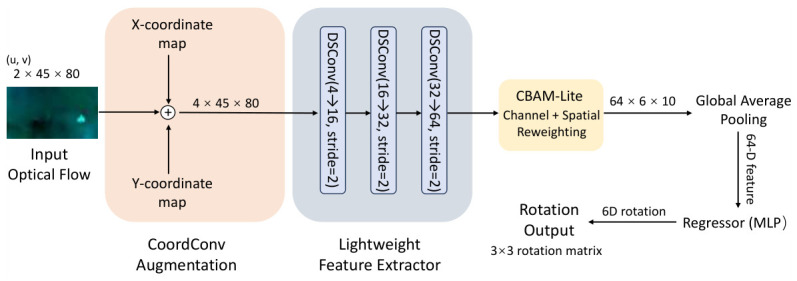
Simplified architecture of the final proposed model. The input low-resolution optical flow is first augmented with normalized spatial coordinate channels through CoordConv. The resulting four-channel tensor is processed by a lightweight three-stage feature extractor based on depthwise separable convolutions, followed by CBAM-Lite refinement and global average pooling. A compact regression head predicts a 6D rotation representation, which is split into two 3D vectors and converted into a valid 3×3 rotation matrix through differentiable Gram–Schmidt orthonormalization.

The architecture of VisualRNet, detailed in Figure 2, is the product of a deliberate design evolution centered on the concept of “specialization.” By focusing solely on the global rotation sub-problem, we obtain a compact model with high parameter efficiency while maintaining competitive accuracy.

#### 3.7.1. Architectural Components and Motivations

- **Explicit Spatial Injection (CoordConv):** Standard convolutional kernels are intrinsically translation-invariant, which can sometimes obscure the center-referenced structure of the global flow field. We utilize **CoordConv** [28] by appending normalized (x,y) coordinate channels (scaled to [−1,1]) directly to the flow input. This introduces an explicit spatial reference and allows the model to anchor its feature extraction to the principal point of the lens. By mapping the input optical flow into a coordinate-dependent representation that respects the center-referenced spatial arrangement of rotational motion, the network can effectively distinguish between identical flow values occurring at different positions, which is essential for determining the precise rotational direction.
- **Non-Linearity for Signed Motion:** Optical flow is a directional vector field, so preserving the distinction between positive and negative responses is important. Standard ReLU activations truncate negative responses to zero and may weaken signed motion cues. We therefore use **LeakyReLU** [29] with a negative slope of 0.1 to preserve sensitivity to both positive and negative responses while avoiding the dead-neuron problem.
- **Factorized Complexity:** To compress the parameter count to the single-digit kilo-scale (K), we replace all standard spatial convolutions with **depthwise separable convolutions** [30]. This factorization decouples the spatial filtering from the channel mixing. Given the relatively compact nature of *u*–*v* optical-flow fields compared to RGB images, this factorization substantially reduces parameters while retaining the capacity to capture the dominant geometric patterns needed for this task.
- **Motion Saliency Refinement:** We integrate a lightweight **CBAM-Lite** module [31] to refine the compact motion features. In our interpretation, this block helps the network reweight informative flow channels and emphasize spatial regions that are more useful for ego-rotation estimation. We avoid stronger causal wording here, because the learned attention is not explicitly supervised with foreground/background labels.

#### 3.7.2. Architecture Summary

VisualRNet processes the augmented 4×45×80 input through three depthwise separable convolution stages (each with a stride of 2), rapidly condensing the field into a 64-channel latent feature map. This is followed by the CBAM-Lite refinement block and a Global Average Pooling (GAP) layer. The final regressor is a purely deterministic Multi-Layer Perceptron (MLP): Linear(64,32)→LeakyReLU→Linear(32,6). We exclude Dropout from the final regression head in the final model variant, because in our experiments this deterministic head yielded slightly better and more stable rotation regression for the present compact setting.

### 3.8. Training Protocol and Experimental Setup

The framework is implemented in PyTorch 2.6.0 with CUDA 12.4. All experiments, including model training, few-shot fine-tuning, and latency evaluation, were conducted on a workstation equipped with a single NVIDIA GeForce RTX 4090 GPU (24 GB VRAM; NVIDIA Corporation, Santa Clara, CA, USA) and an Intel Core i9 processor (Intel Corporation, Santa Clara, CA, USA).

We follow the current training implementation. The model is optimized with AdamW, an initial learning rate of $1\times 10^{-3}$, weight decay of $1\times 10^{-4}$, and batch size 64. We use four data-loading workers, automatic mixed precision, gradient clipping with max_norm =1.0, and a cosine-annealing schedule with $T_{\max}=300$ and $\eta_{\min}=1\times 10^{-6}$. No early stopping is used; all runs are trained for a maximum of 300 epochs. The checkpoint with the lowest validation loss is retained for final testing.

For model development, we partition the official Deep-FVS training portion into training and validation subsets, using two thirds for optimization and one third for validation. This yields 9186 training samples, 4593 validation samples, and 19,363 test samples in the official test split. Unless otherwise specified, the reported geometric metrics are angular errors in degrees.

We additionally report inference FLOPs per frame pair for the compared learning-based methods. Unless otherwise specified, FLOPs are measured for the full inference model at the evaluation input resolution and include both the optical-flow frontend and the regression backend.

Latency and FPS are measured with batch size 1 on a single RTX 4090 GPU, using 50 warm-up iterations followed by 200 timed runs with CUDA synchronization. Reported latency is the mean wall-clock time per frame pair in milliseconds, and FPS is computed as 1000/latency(ms). In Table 1, the reported parameter count, FLOPs, latency, and FPS for the learning-based methods correspond to the full model used at inference. The head-only cost of VisualRNet is reported separately in Table 2.

## 4. Results

### 4.1. Internal Design Comparison

Before comparing against external baselines, we first examine the internal design path of the proposed regression backend. Table 3 compares the three backend variants evaluated in this paper: a plain convolutional baseline, a lighter variant that combines depthwise separable convolutions with CBAM-Lite, and the final VisualRNet head. Because all three variants share the same optical-flow frontend, the parameter count, latency, and FPS reported in this table correspond to the regression backend only. This presentation makes the lightweight design progression easier to interpret without mixing in the constant frontend cost.

Table 3 shows that even the plain convolutional baseline already achieves sub-degree mean error, indicating that low-resolution optical flow preserves enough global structure for frame-to-frame rotation regression. The DWConv + CBAM-Lite variant provides a strong intermediate operating point with the smallest backend and the highest accuracy–efficiency ratio among the lightweight alternatives, while the final VisualRNet head further improves RMSE and P95 at only a modest backend cost increase.

### 4.2. Full-Pipeline Comparison with External Baselines

To position the proposed method against external baselines, Table 1 compares the full VisualRNet pipeline with a classical geometric baseline and a representative RGB-based visual-odometry model. In this table, the reported parameter count, FLOPs, latency, and FPS correspond to the full model used at inference.

### 4.3. Transfer Evaluation of TartanVO on Deep-FVS

To provide a direct transfer comparison with a representative RGB-based learning baseline, we reproduced TartanVO under the same Deep-FVS rotation-only evaluation protocol. TartanVO was originally developed for visual odometry rather than for the specific task of camera rotation estimation considered here. We therefore use it as a representative transfer baseline and evaluate its out-of-the-box rotation estimation capability on Deep-FVS without task-specific retraining. This comparison is intended as a representative reference rather than an exhaustive benchmark over all recent VO architectures.

Concretely, TartanVO takes two consecutive RGB frames as input and outputs relative camera motion, from which the relative rotation component is extracted for evaluation. The reference relative rotation is constructed by integrating the gyroscope angular velocity signal provided by Deep-FVS over the corresponding interval between adjacent frame timestamps. We then compute the geodesic angular error between the predicted rotation and this IMU-derived reference rotation, and summarize the results with Mean, Median, RMSE, and P95. Parameter count, FLOPs, latency, and FPS are reported as efficiency indicators.

The reproduced TartanVO result is included in Table 1. On Deep-FVS, TartanVO reaches a mean error of $0.8040^{\circ }$ with a 24.51 M-parameter, 107.714 GFLOPs full model running at 31.56 FPS, whereas VisualRNet reaches $0.3151^{\circ }$ with a 1.374 M-parameter, 7.194 GFLOPs full pipeline running at 112.99 FPS. Since TartanVO is not specifically optimized for the present task or dataset, this comparison is primarily intended to reflect the transferability of a representative RGB-based VO model to camera rotation estimation. This result should not be read as implying that TartanVO is inferior as a general visual-odometry model; rather, it indicates that a broader RGB-based odometry model does not automatically provide a better cost–accuracy trade-off on a narrower rotation-sensing benchmark. At the same time, the result supports the intended positioning of VisualRNet: when the downstream requirement is limited to frame-to-frame rotation sensing, a lightweight motion-centric pipeline can offer a more favorable cost–accuracy trade-off than a broader RGB-based odometry model.

### 4.4. Cumulative Rotation Comparison

**Figure 3:**
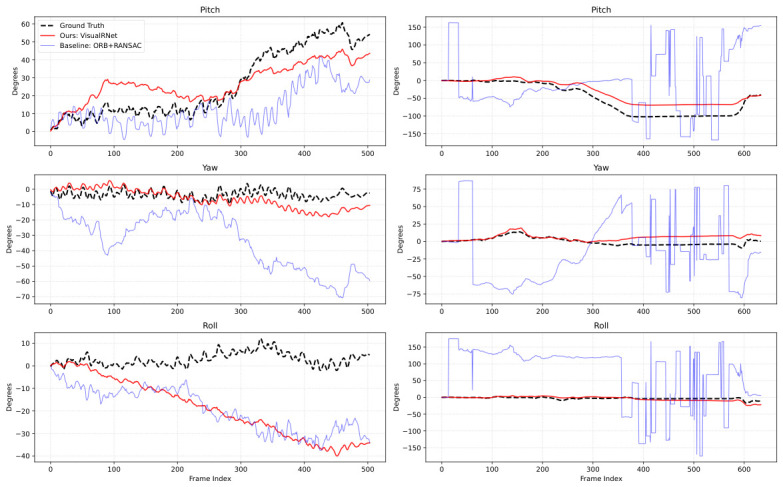
Cumulative rotation comparison on two representative sequences. The left column shows a relatively simple outdoor sequence, and the right column shows a challenging indoor nighttime sequence. In each subplot, the dashed black curve denotes the IMU-integrated reference trend, the red solid curve denotes VisualRNet, and the blue solid curve denotes ORB + RANSAC. VisualRNet tracks the reference more closely in both cases, with a larger advantage in the challenging indoor sequence.

To visualize the practical implications of these quantitative results on long-term stability and drift accumulation, we continuously integrate the frame-to-frame predictions into an absolute camera trajectory. Figure 3 showcases this trajectory comparison on two representative video sequences featuring distinct environmental challenges.

In the relatively simpler outdoor sequence (Figure 3, left), VisualRNet tracks the IMU-integrated reference trajectory more closely than the ORB + RANSAC baseline. The gap becomes larger in the indoor nighttime sequence (Figure 3, right), where the classical geometric method suffers from repeated feature-matching failures under low illumination and blur. VisualRNet remains substantially more stable in this example, suggesting that the motion-centric representation is less sensitive to these degradations.

### 4.5. Instantaneous Relative Rotation Comparison

**Figure 4:**
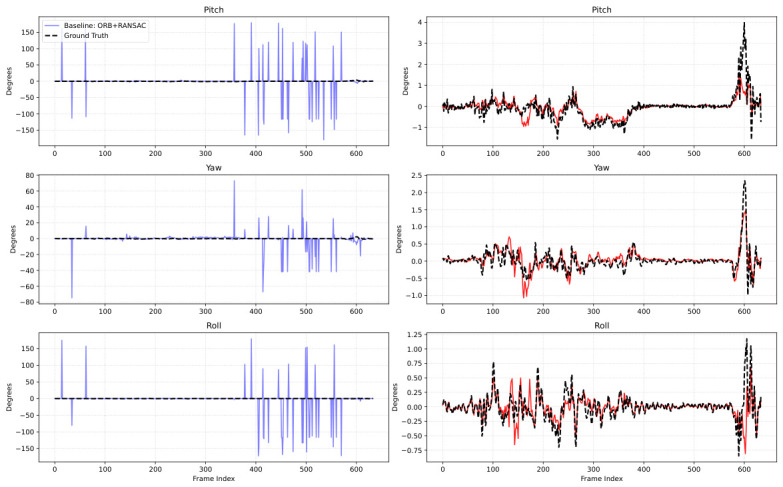
Instantaneous relative rotation comparison on a representative challenging sequence. The left column shows the ORB + RANSAC baseline, and the right column shows VisualRNet. In all subplots, the dashed black curve denotes the IMU-integrated reference trend, while the colored solid curve denotes the corresponding method prediction. Across pitch, yaw, and roll, the ORB + RANSAC estimates exhibit frequent spikes, whereas VisualRNet follows the reference trend more closely.

To further examine short-term responsiveness and high-frequency rotational tracking, we compare the instantaneous frame-to-frame rotation predictions on a representative challenging sequence. Figure 4 presents the pitch, yaw, and roll curves for the ORB + RANSAC baseline and for VisualRNet, together with the IMU-integrated reference trend.

The temporal plots reveal that the ORB + RANSAC estimates are noisy and occasionally unstable, while VisualRNet follows the IMU-integrated reference trend more closely over time. These examples are consistent with the quantitative results, although they should be interpreted as representative case studies rather than exhaustive proof of broad domain robustness.

### 4.6. Module-Level Ablation Study

To validate the architectural choices in Section 3.7, we conduct an ablation study summarized in Table 4. CoordConv and LeakyReLU are kept active across all compared variants, so the table focuses on the incremental effects of depthwise separable convolutions, CBAM-Lite, and the final regression head. Because all variants share the same FastFlowNetv2 frontend, the reported parameter counts in Table 4 correspond to the regression backend only; the differences across rows therefore isolate the backend design changes. The results show that progressively simplifying the feature extractor and refining the regression head improves the accuracy–efficiency balance for this rotation-specific task.

### 4.7. Cross-Camera Adaptation with Lightweight Calibration

Beyond the in-domain benchmark, we also test whether the learned motion representation can be re-aligned to a different camera setup with limited target-domain data. This is a narrower and more defensible question than broad cross-domain transfer: the goal is not zero-shot transfer, but rapid camera-specific calibration when the sensor intrinsics or imaging characteristics change.

To test cross-camera adaptability, we perform a lightweight calibration experiment on the unseen TUM VI dataset [32], whose camera intrinsics and sensor setup differ from those of Deep-FVS. We initialize the model from Deep-FVS pre-training and use the first 500 frames of a TUM VI sequence as a compact adaptation set.

During this adaptation stage, we freeze the convolutional backbone and CBAM-Lite module and fine-tune only the final MLP regression head. This isolates the question of whether the motion representation learned on Deep-FVS can be re-aligned to a new camera with limited target-domain data.

As reported in Table 5, the adapted model achieves a mean relative pose error of 0.7072° and an RMSE of 0.8424° on 5489 unseen test frames. We interpret this experiment as evidence of practical cross-camera adaptability rather than broad zero-shot transfer: the results suggest that the learned motion representation can be transferred to a new camera system with modest calibration effort, but they do not by themselves establish universal transfer across domains.

### 4.8. Robustness to Noisy Optical Flow

In practical deployment, the optical flow estimated by the frontend is not perfect. To evaluate the sensitivity of VisualRNet to degraded motion input, we inject additive Gaussian noise into the optical-flow tensors at test time. Specifically, the corrupted flow is generated as $F_{\mathrm{noise}}=F+N\left(0,\sigma^{2}\right)$, where σ controls the perturbation level.

VisualRNet is evaluated under five noise levels, from σ=0.0 to σ=0.5. Table 6 reports the mean, median, standard deviation, maximum angular error, and two success rates (<5° and <1°).

VisualRNet remains relatively stable under this synthetic perturbation. As the noise level increases from σ=0.0 to σ=0.5, the mean error rises from $0.3149^{\circ }$ to $0.5212^{\circ }$, while the success rate below $5^{\circ }$ remains at 100.00%. These results suggest that the model relies primarily on global motion structure rather than local flow perturbations. At the same time, this experiment should be interpreted as controlled stress testing rather than a complete substitute for evaluating all real-world failure modes of optical-flow estimation.

## 5. Discussion

The results of this study support a narrower but practically useful conclusion: for frame-to-frame rotational sensing, low-resolution optical flow can retain enough global structure to enable accurate regression. This finding is relevant to stabilization-oriented applications, where rotational jitter is especially important and where a compact motion representation may be preferable to a heavier RGB pipeline.

This positioning is important relative to broader visual geometry pipelines. Recent systems such as DUSt3R, MASt3R, VGGT, and Align3R estimate richer quantities—for example camera parameters, dense point maps, depth, multi-view tracks, or temporally aligned video geometry—from uncalibrated or weakly calibrated imagery [11,12,13,14]. These methods are valuable when the downstream objective is scene reconstruction or general 3D reasoning. Our goal is different: we deliberately trade breadth for cost and simplicity. If a downstream module mainly needs the rotational trend and magnitude, then solving the larger problem of scene reconstruction can be unnecessary overhead. In that sense, VisualRNet should not be read as a reduced version of full 3D geometry systems, but as a task-level compromise aimed at a lower-cost operating point. We do not question the value of broader VO, SLAM, or feed-forward geometry systems; our point is only that when the downstream requirement is limited to approximate frame-to-frame rotational trend and magnitude, solving the larger geometry problem may incur unnecessary modeling and deployment costs.

A second contribution of the paper lies in the design path of VisualRNet. Rather than presenting a single opaque architecture, we show how coordinate injection, lightweight convolutional design, CBAM-Lite refinement, and a compact 6D regression head affect the final accuracy–efficiency trade-off. In this sense, the paper is as much about task-specific model design as it is about the final numerical result.

At the same time, the scope of the present study should be stated clearly. VisualRNet is designed for rotation estimation rather than full 6 DoF motion. Its performance is therefore bounded not only by the quality of the optical-flow frontend, but also by the degree to which translational motion, scene depth variation, and independently moving objects perturb the global flow structure. These factors deserve more systematic study in future work.

The few-shot TUM VI experiment further suggests that the learned representation can be adapted to a new camera system with limited calibration data. We view this as promising practical transferability, not as evidence of universal domain invariance. Future work will investigate stronger cross-dataset evaluation, integration with temporal models, and possible extensions from rotation-only sensing to lightweight 6 DoF estimation.

## 6. Conclusions

This paper presented VisualRNet, a lightweight framework for estimating frame-to-frame camera rotation from low-resolution optical flow under cross-modal IMU supervision. The experiments show that low-resolution motion fields can serve as an effective input for rotation regression, and that a compact coordinate-aware network can achieve competitive accuracy on Deep-FVS while remaining computationally efficient at the regression-head level.

We further clarified the scope and interpretation of the results. The reported 7.7 K parameters, 0.002 GFLOPs, and 729 FPS correspond to the VisualRNet regression head, while the end-to-end pipeline additionally includes the FastFlowNetv2 frontend and reaches 1.374 M parameters, 7.194 GFLOPs, and approximately 113 FPS on our hardware. The TUM VI adaptation experiment indicates practical cross-camera adaptability after limited calibration, but should not be interpreted as broad zero-shot transfer.

Overall, the study supports a more modest but practically meaningful conclusion: for stabilization-oriented rotational sensing, a compact motion-centric network can provide reliable frame-to-frame rotation estimates without solving the broader RGB-based full-odometry problem. The reproduced TartanVO result further reinforces this positioning: a much larger general-purpose VO model does not necessarily provide a better cost–accuracy trade-off for this narrower benchmark. The contribution is therefore not to replace full 3D geometry or full visual odometry, but to show that a narrower rotation-sensing objective can be solved at substantially lower modeling and deployment cost when only rotational trend and magnitude are required by the downstream task. Future work will investigate stronger cross-dataset validation, a more systematic treatment of translational interference and dynamic objects, and extensions toward lightweight full-pose estimation.

## Figures and Tables

**Table 1:** Full-pipeline comparison on the Deep-FVS test set. For the learning-based methods, the reported parameter count, FLOPs, latency, and FPS correspond to the full model used at inference. For VisualRNet, the head-only cost is reported separately in Table 2.

Method	Mean	Median	RMSE	P95	Params (Full Model)	FLOPs (Full Model)	Latency (ms, Full Model)	FPS (Full Model)
ORB + RANSAC	38.6767	0.9442	82.5208	156.3000	N/A	N/A	15.000	66.67
TartanVO [5]	0.8040	0.5056	1.2046	2.6997	24.51 M	107.714 G	31.688	31.56
**VisualRNet (Ours)**	**0.3151**	**0.2221**	**0.4393**	**0.9018**	**1.374 M**	**7.194 G**	**8.850**	**112.99**

**Table 2:** Cost decomposition of the proposed system. The reported 7.7 K parameters and 729.39 FPS correspond to the VisualRNet regression head only; the full pipeline additionally includes the FastFlowNetv2 frontend. FLOPs are reported to make the head-only and full-pipeline computational cost more directly comparable.

Component	Params	FLOPs	Latency (ms)	FPS
FastFlowNetv2 frontend	1.366 M	7.192 G	7.479	133.71
VisualRNet head	7.7 K	0.002 G	1.371	729.39
**Full pipeline**	**1.374 M**	**7.194 G**	**8.850**	**112.99**

**Table 3:** Internal comparison of the proposed backend design on the Deep-FVS test set. Since all variants share the same FastFlowNetv2 frontend, the reported parameter count, latency, and FPS correspond to the regression backend only.

Backend Variant	Mean	Median	RMSE	P95	Params (Backend)	Latency (ms, Backend)	FPS (Backend)
Plain convolutional baseline	0.3349	0.2357	0.4681	0.9787	25,942	**0.715**	**1397.98**
DWConv + CBAM-Lite variant	0.3158	0.2238	0.4486	0.9273	**5642**	0.854	1171.34
**VisualRNet (final backend)**	**0.3151**	**0.2221**	**0.4393**	**0.9018**	7740	1.371	729.39

**Table 4:** Module-level ablation study mapping the progression to VisualRNet. Since all configurations share the same FastFlowNetv2 frontend, the reported parameter counts correspond to the regression backend only.

Configuration	DW Conv	CBAM-Lite	Enhanced Head	Mean	RMSE	Params (Backend)
Plain convolutional baseline	–	–	–	0.3349	0.4681	25,942
Baseline + DW Conv	✓	–	–	0.3335	0.4660	6021
+ CBAM-Lite (DWConv + CBAM-Lite variant)	✓	✓	–	0.3158	0.4486	**5642**
+ Enhanced Head (VisualRNet)	✓	✓	✓	**0.3151**	**0.4393**	7740

**Table 5:** Few-shot camera-specific adaptation results of VisualRNet on the unseen TUM VI dataset. Evaluated strictly on 5489 unseen testing frames after fine-tuning only the MLP head on the initial 500 frames.

Setting	Mean RPE (deg)	Median RPE (deg)	RMSE (deg)
Deep-FVS in-domain baseline test	0.3151	0.2221	0.4393
TUM VI **Few-Shot Adaptation Test**	**0.7072**	**0.6200**	**0.8424**

**Table 6:** Robustness of VisualRNet under escalating Gaussian noise levels explicitly added to the input optical flow tensors during the inference phase.

Noise (σ)	Mean (deg)	Median (deg)	Std (deg)	Max (deg)	Success $<5^{\circ }$	Success $<1^{\circ }$
0.0	0.3149	0.2220	0.3064	3.6599	100.00%	96.46%
0.1	0.3543	0.2644	0.2952	3.6104	100.00%	96.27%
0.2	0.4259	0.3520	0.2932	3.5789	100.00%	95.41%
0.3	0.4649	0.3983	0.2969	3.7548	100.00%	94.74%
0.5	0.5212	0.4523	0.3150	3.5762	100.00%	92.90%

## Data Availability

The Deep-FVS dataset used in this study is publicly available. Source code for VisualRNet and trained model weights will be made available upon acceptance or can be provided by the corresponding author upon reasonable request.

## Abbreviations

CNN	Convolutional Neural Network
FPS	Frames Per Second
IMU	Inertial Measurement Unit
ORB	Oriented FAST and Rotated BRIEF
P95	95th-Percentile Error
RANSAC	Random Sample Consensus
RMSE	Root Mean Square Error
SLAM	Simultaneous Localization and Mapping
